# Vortioxetine alleviates motor, cognitive and emotional disorders in post-stroke rats by regulating the TLR-2/NF-*κ*B pathway

**DOI:** 10.3389/fphar.2025.1555079

**Published:** 2025-03-12

**Authors:** Ziqiang Dong, Zhihui Dong, Lili Xu, Jinfeng Zhang, Lin Li, Rongjuan Wang, Xiaoyan Huang, Zhengqiang Zou

**Affiliations:** ^1^ Department of Anesthesiology, Ganzhou Hospital-Nanfang Hospital, Southern Medical University, Ganzhou, China; ^2^ Department of Planning and Quality Control, Ganzhou Hospital-Nanfang Hospital, Southern Medical University, Ganzhou, China; ^3^ Intravenous Drug Dispensing center, Ganzhou Hospital-Nanfang Hospital, Southern Medical University, Ganzhou, China; ^4^ Ganzhou Rongjiang New Area People’s Hospital, Ganzhou, China

**Keywords:** vortioxetine, ischemic stroke, cognitive dysfunction, neuroinflammation, TLR-2/NF-κB pathway

## Abstract

Cognitive impairments following post-stroke significantly hinder neurological recovery and exacerbate patient morbidity, underscoring urgent need for effective therapeutic strategies. Vortioxetine (VTX), a prominent Selective Serotonin Reuptake Inhibitor (SSRI), boasts notable antidepressant, cognition-enhancing, and anti-inflammatory properties. This investigation delves into VTX’s influence on motor skills, spatial learning-memory capabilities, and depressive behaviors in Middle Cerebral Artery Occlusion (MCAO) rats, alongside its underlying mechanisms. Our findings reveal that while VTX fails to entirely reverse ischemic-reperfusion damage, it substantially ameliorates spontaneous locomotor functions, augments post-stroke learning-memory capacities, and exhibits potent antidepressant and anxiety-like efficacy. Preliminary data propose that these beneficial effects may stem from inflammation modulation via the Toll-Like Receptor 2 (TLR-2)/Nuclear Factor-Kappa B (NF-*κ*B) signaling pathway. Collectively, our work underscores VTX’s promising role in enhancing motor, cognitive functions, and mitigating depressive symptoms following cerebrovascular accidents, potentially through inflammation regulation. These insights pave the way for novel interventions addressing post-stroke complications, warranting further exploration.

## Introduction

Ischemic stroke which may be caused by a narrowing or blockage of the brain vessel, is a common cerebrovascular disease with high morbidity, mortality and disability (Galligan et al., 2016; Benjamin et al., 2017). According to statistics, 20% of post-stroke cognitive impairment can develop into dementia, especially within 1 year after stroke the risk is higher (Schneider and Schneider, 2012). Cognitive dysfunction primarily involves impairments in patients’ memory, executive function, spatial perception, calculation ability, attention, and orientation. These deficits can gradually erode patients’ capacity for independent living, imposing a substantial burden on both their families and society. Studies have shown that 50∼75% of stroke patients are accompanied by cognitive dysfunction such as learning, memory and language (Johnston et al., 2024; Zhou et al., 2024). The current treatment mainly involves emergency treatment in the acute phase and continuous nursing during the long-term rehabilitation period to help patients regain normal function and improve their quality of life to the greatest extent possible. However, despite some progress, several challenges remain in the treatment of ischemic stroke, including inadequate understanding of the causes, inadequate preventive measures, and uneven distribution of rehabilitation services. Tt is very necessary to deepen the research on cognitive dysfunction after stroke, formulate corresponding treatment plans, and improve the cognitive dysfunction of patients.

Neuroinflammatory response is one of the important mechanisms of ischemia-reperfusion injury, which involves the activation and release of various inflammatory cells and mediators. Notably, pro-inflammatory responses are not exclusive to post-stroke pathology but are also a hallmark of depression, with elevated levels of cytokines such as IL-6 and TNF-α observed in depressive patients. Importantly, effective antidepressant therapies, including pharmacological interventions, have been shown to normalize these inflammatory parameters, suggesting a bidirectional relationship between neuroinflammation and psychiatric disorders (Dantzer et al., 2008). Inflammatory cytokines such as IL-1β, IL-6, and TNF-ɑ increased within 24 h, peaked 2∼3d, and remained at high levels for several months after stroke onset (Sun et al., 2024; Yang et al., 2019). In this process, toll-like receptor 2 (TLR-2) and nuclear factor *κ*B (NF-*κ*B) play important regulatory roles (Tamai et al., 2020; Zou et al., 2017). TLR-2 is a receptor protein that recognizes molecular patterns of pathogens and activates immune responses. Activation of TLR-2 in ischemia-reperfusion injury is considered to be one of the key steps in triggering neuroinflammatory response. Once TLR-2 is activated, it initiates a series of signal transduction pathways that ultimately lead NF-*κ*B activation. NF-*κ*B is a crucial transcription factor facilitating the transcription and expression of various inflammatory genes, including cell adhesion molecules, inflammatory mediators, and apoptosis-related genes. The TLR-2/NF-*κ*B pathway has a significant impact on ischemia-reperfusion injury, and it may be a potential therapeutic target for inhibiting neuroinflammatory response and mitigating injury.

Vortioxetine is a multimodal-acting antidepressant uniquely positioned to address post-stroke complications due to its combined anti-inflammatory and neurorestorative properties. Beyond its serotonergic modulation, preclinical evidence indicates that vortioxetine suppresses pro-inflammatory cytokines (e.g., IL-6, TNF-α) through 5-HT3 receptor antagonism and enhances synaptic plasticity via 5-HT1A receptor agonism, both mechanisms implicated in post-stroke recovery (Krupa et al., 2023). These receptor-mediated actions are particularly relevant for cognitive enhancement and neuroplasticity, as demonstrated in preclinical models. Critically, vortioxetine’s ability to simultaneously target neuroinflammation and synaptic remodeling aligns with the dual pathophysiology of post-stroke cognitive and affective disorders. This mechanistic synergy provides a strong rationale for investigating its effects on the TLR-2/NF-*κ*B pathway, a key driver of inflammatory cascades in cerebral ischemia. Studies have found that vortioxetine can reduce the activation and infiltration of inflammatory cells, reduce the production of cytokines, and thus relieve the symptoms of neuroinflammation (Alboni et al., 2021). In studies of ischemic stroke, vortioxetine has been found to have neuroprotective effects (Emam et al., 2021). It can mitigate the injury caused by cerebral ischemia by increasing the concentration of serotonin in brain tissue, improving the survival rate of nerve cells, reducing apoptosis and oxidative stress response. Moreover, vortioxetine can not only boost the proliferation and differentiation of neural stem cells but also facilitate the repair and regeneration of brain tissue. The broad clinical applicability of vortioxetine is further supported by its favorable safety profile, which allows its use in diverse patient populations including those with epilepsy, somatic comorbidities, bipolar depression, and dementia, as evidenced by recent clinical observations (Gamberini et al., 2021; Fagiolini et al., 2021).

Central and peripheral inflammatory responses occur after ischemic stroke, in which immune cells in brain tissue such as microglia, astrocytes, and oligodendrocytes are activated and release cytokines, chemokines, and secondary inflammatory mediators (Nilupul et al., 2006). Neutrophil recruitment took place within the initial 24 h following the onset of stroke., and macrophage recruitment occurred 3 days later. Inflammatory response can bring about changes in emotional and cognitive functions linked to depression. Studies have shown that antidepressants can reduce the level of inflammatory cytokines, improve cognitive dysfunction in patients with post-ischemic stroke, and improve patients’ ability to live independently (Tomaz et al., 2020; Yu et al., 2018; Zhang and Bai, 2022). VTX has an antidepressant effect by inhibiting central serotonin (5-HT) reuptake and enhancing 5-HT activity. Studies have also shown that VTX can improve the learning and memory ability of chronically stressed mice by inhibiting central nervous system inflammation (Nemutlu et al., 2022). Emerging evidence suggests vortioxetine may improve post-stroke cognitive outcomes, as demonstrated in a recent clinical study showing its efficacy in reducing cognitive impairment following cerebrovascular events (Gamberini et al., 2021; Emam et al., 2021). In this project, the cerebral ischemia reperfusion model of right middle cerebral artery occlusion in SD rats was prepared by modified Longa-Zea’s suture method, the impact and mechanism of VTX on the cognitive function of rats with ischemic stroke were explored, providing a new treatment method and approach for the recovery of neurological function after cerebral ischemia.

## Materials and methods

### Study design

**FIGURE 1:**
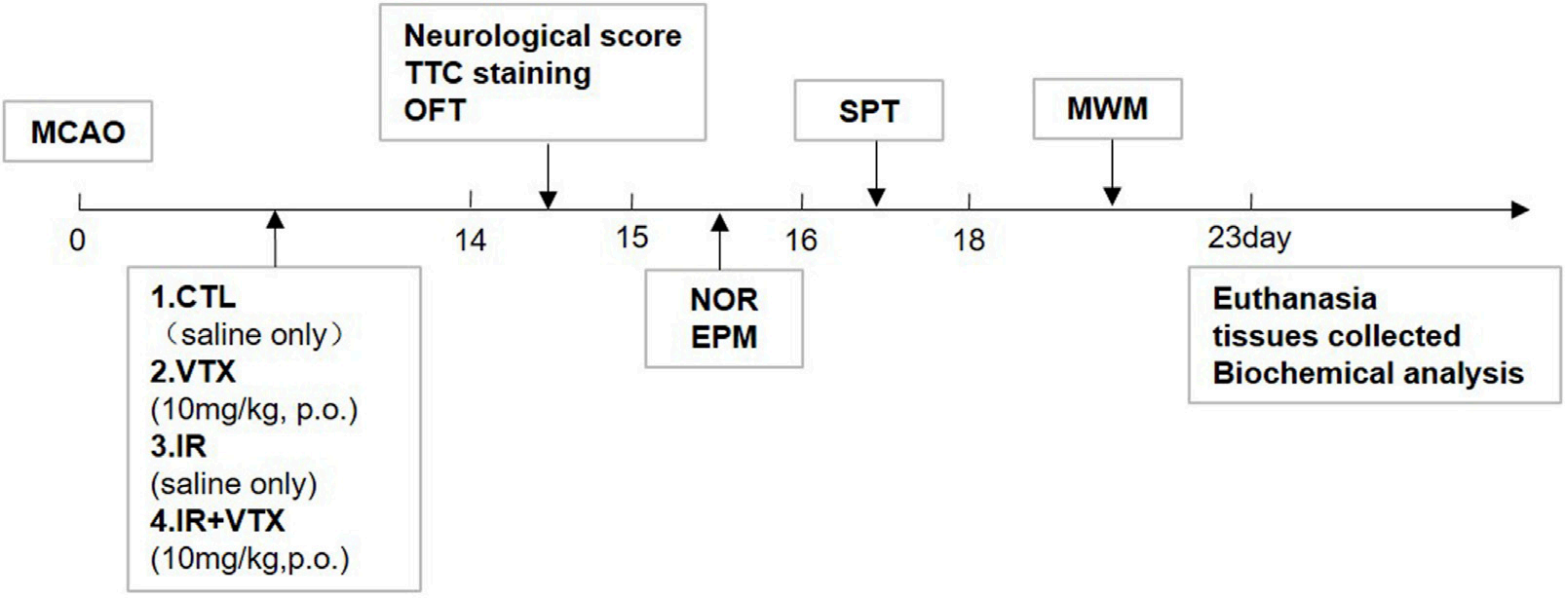
Schedule of the study design including MCAO model establishment, VTX dosing, cerebral infarction analysis, neurological scoring and behavioral tests, biochemical analysis.

The animals were randomly allocated into four groups:

1) Control (CTRL) group: After undergoing a sham procedure (where the carotid arteries were exposed for 45 min, yet no ischemia was induced), the rats were administered distilled water for 14 days; 2) VTX group: Rats underwent a sham operation and received VTX for 2 weeks; 3) Ischemia-reperfusion (IR) group: Rats underwent MCAO for 45 min and received distilled water for 2 weeks; 4) IR + VTX group: Rats underwent MCAO for 45 min and received VTX for 2 weeks (Figure 1). This study was approved by the Animals Ethics Committee of Southern Medical University, and all procedures were performed in accordance with the Guide for the Care and Use of Laboratory Animals. All surgical procedures were performed under isoflurane anesthesia (induction 5%, maintenance 2%), and postoperative analgesia (buprenorphine, 0.05 mg/kg subcutaneously every 12 h for 48 h) was administered to minimize discomfort. This study adhered to the ARRIVE guidelines for reporting animal research.

### Animals

Healthy adult male Sprague–Dawley rats, aged 14–16 weeks and weighing 280–320 g, were procured from the Laboratory Animal Centre of Southern Medical University (Guangzhou, China). Prior to the experiments, these rats were allowed to acclimate in the facility for 1 week. Six rats were placed in each cage and kept under standard environmental conditions: a temperature of 22°C ± 2°C, a relative humidity of 60% ± 5%, and a 12 - hour light/dark cycle where the lights came on at 7:00. The rats had unrestricted access to food and water. The experimental procedures were carried out between 8:30 and 17:30. All experimental procedures were performed in compliance with local ethical guidelines for animal research. Before the experiments commenced, they were approved by the relevant government agency or the institutional animal care and use committee.

### Drugs

VTX was purchased from Sigma-Aldrich Chemical Co. (St. Louis, MO, United States). Before use, VTX was freshly dissolved in distilled water. It was then administered orally for 2 weeks at a dose of 10 mg/kg/day.

### Establishment of the MCAO model

The surgical procedures for middle cerebral artery occlusion and reperfusion (MCAO/R) were described previously (Longa et al., 1989). Briefly, the rats were anesthetized via intraperitoneal injection of 10% chloral hydrate at a dose of 300 mg/kg. Subsequently, the left common carotid artery, external carotid artery, and internal carotid artery were meticulously isolated through blunt dissection. Next, a nylon monofilament (Cinontech, Beijing, China) with a blunt tip was advanced up to 18–20 mm from the carotid bifurcation to block the origin of the middle cerebral artery. 45 min after ischemia was induced, the filament was gently retracted to restore blood perfusion. Throughout the operation and the period of ischemia, the body temperature of the animals was kept at 37.0°C ± 0.5°C using a heating pad. Rats in the sham group underwent an identical surgical procedure, except that no filament was inserted.

### Neurological deficit scores assessment

On the 14th day following the surgery, the neurological deficit score was assessed by researchers who were unaware of the animal groupings. A score of 0 was assigned when no obvious neurological deficit was observed. If the contralateral forepaw could not be extended when the rat was lifted by the tail, it was scored 1. Circling towards the contralateral side while maintaining a normal resting posture was scored 2. A severe focal deficit, such as falling to the contralateral side, was scored 3. Inability to walk independently along with a reduced level of consciousness was scored 4. Rats that received a score of 0 after ischemia-reperfusion (I/R) surgery were considered failed models and were removed from subsequent experiments.

### Infarct volume assessment

Infarct volume was assessed using 2,3,5 - triphenyltetrazolium chloride (TTC, purchased from BBI Life Sciences, Shanghai, China) staining. Briefly, 2 weeks after reperfusion, the animals were euthanized and their brains were quickly removed. After being rinsed with cold saline, the brain tissues were placed at −20°C for 30 min. Frozen brain samples were cut into sections (2 mm thick), which were then immersed in 2% TTC solution at 37°C for 30 min in the dark. Following fixation with 4% paraformaldehyde, the brain sections were photographed. The images were analyzed with Image - Pro Plus 6.0 software. Percentage of infarct volume was calculated as follows: infarct volume (%) = (contralateral section area – noninfarcted ipsilateral section area)/contralateral section area × 100%.

### Behavioral tests

Behavioral tests were conducted by a blinded investigator at 2 weeks after MCAO induction.

#### Open field test (OFT)

Locomotor activity was measured in an open - field setup. The apparatus consisted of a black plastic board measuring 80 × 80 cm, enclosed by black plastic walls that were 40 cm tall. Each rat was put into the arena and allowed to explore for 5 min. The movements were recorded by a video tracking system (Noldus Ethovision XT System, the Netherlands). Total distance traveled, mean velocity, and the total number of square crossings were calculated (Zhou et al., 2018).

#### Novel object recognition (NOR) test

The test was conducted as previously described (Jiang et al., 2020). On day 15, each rat was permitted to move freely in a white box (80 × 80 × 40 cm) for 5 min to acclimate to the environment. Twenty - four hours later, the rats were individually positioned in the center of the box. Inside the box, two identical objects were placed in diagonally opposite corners. The time each rat spent exploring each object was recorded over a 5 - minute period. Exploration was defined as touching the object or facing it within a 2 - cm range. For the memory test, the same procedure was followed, except that one of the objects was replaced with a new object that differed in shape and color. The recognition index was expressed by the ratio TN/(TF + TN), where TF = time spent exploring the familiar object and TN = time spent exploring the novel object.

#### Morris water maze (MWM) test

Spatial learning and memory abilities were tested with the Morris water maze (MWM) as described previously (Jiang et al., 2020). The test was carried out using the black circle pool filled with water (22°C ± 1°C), which was divided into four quadrants (I, II, III, and IV). To make it invisible to rats under the experimental light, the escape platform was located 2 cm below the water surface in the northeastern (NE) quadrant. The rats were subjected to the location navigation experiment on days 1–4: the rats were placed into the water facing the pool wall in a counterclockwise direction from four quadrants, observed, and timed for 60s. The escape latency, measured as the time the rat spent searching for and climbing onto the platform, was recorded using a camera system, and rats were guided to the platform and allowed to stay for 15 s if they did not find the platform within 60s, the escape latency was measured as 60s. The learning ability of the rats was reflected after completion with an escape latency period of 4 days. The spatial exploration experiment was conducted on the rat’s 5th day: the flat withdrawal, the rat was placed into the water facing the wall of the pool from the I quadrant farthest from the original platform, and the swimming time of each quadrant in 60s was recorded, with the spatial exploration time to swim in the II quadrant where the original platform was located, as memory performance.

#### Sucrose preference test (SPT)

Depression-like behavior was tested with a 48 h SPT. Experiments were performed as previously described (Yu et al., 2018). On day 1, the rats were allowed to get used to drinking from two bottles, each filled with a 5% sucrose solution. On day 2, one of the bottles was swapped out for a bottle filled with pure water. Before a 12 - hour testing period, the rats were deprived of both water and food for 24 h. Sucrose preference was defined as (weight of sucrose ingested)/(weight of water ingested + weight of sucrose ingested) × 100.

#### Elevated plus maze (EPM) test

The anxiety - like behaviors of rats were assessed using an elevated plus - maze (EPM) apparatus. The apparatus had two open arms, each measuring 50 × 10 cm, which intersected with two closed arms. The closed arms were surrounded by 40 - cm - high walls, and the entire structure was elevated 50 cm above the ground. The method used was the same as previously described (Kokare et al., 2005). The rats were individually placed at the center, facing an open arm, and the animal’s behavior was monitored by an overhead video tracking system (Noldus Ethovision XT System, Netherlands). The time percentage spent in open and closed arms, the entry percentage of open and closed arms, and the anxiety index were computed. To avoid bias caused by the scent of the previous rat, the maze was cleaned with 70% alcohol between each trial. The calculation formula for the anxiety index is as follows:

$$\text{Anxiety index}=1-\left[\left(\left[\frac{\text{Open}\;\text{arm}\;\text{time}}{\text{Test}\;\text{duration}}\right]+\left[\frac{\text{Open}\;\text{arm}\;\text{entries}}{\text{Total}\;\text{number}\;\text{of}\;\text{entries}}\right]\right)÷2\right]$$

### Western blot analysis

Protein expression levels were determined in the hippocampal and cortical tissues of rats. Western blotting analysis was performed following previously reported procedures, with minor adjustments. Brain tissues were homogenized with the Radio Immunoprecipitation Assay (RIPA) lysis buffer (including 1% protease inhibitor cocktail, 1% phosphatase inhibitor cocktail) containing a protease inhibitor cocktail (Sigma-Aldrich), centrifuged at 12,000× g for 10 min, BCA protein assay kit (Thermo Scientific, Waltham, MA, United States) was used to measure the total protein concentration, and equal amounts of protein were separated by sodium dodecyl sulfate polyacrylamide gel electrophoresis. Protein bands were transferred to PVDF membranes, and incubated with anti-NF-kB p65 (1:1000, 8242T, Cell Signaling Technology), rabbit antibody against TLR-2 (1:1000, ab209217, Abcam, Cambridge, MA, United States), or rabbit GAPDH (1:10,000, ab181602, Abcam, Cambridge, MA, United States) and anti-histone H3 (Cell Signaling Technology) antibodies overnight at 4°C, then incubated with goat anti-rabbit IgG horse radish peroxidase (1:5000, 98164S, Cell Signaling Technology, Danvers, MA, United States) for 2 h at room temperature. The bands were quantified with ImageJ.

### Enzyme-linked immunosorbent assay

The levels of inflammatory cytokines and NFκB-p65 were quantified using enzyme-linked immunosorbent assay (ELISA) kits. Commercially available ELISA kits (Elabscience, MD, United States) for rat TNF-α (E-ELR0019), IL-1β (E-EL- R0012), IL-6 (E-EL- R0015), and NF*κ*B-p65 (ab176647; Abcam, Cambridge, MA, United States) were performed according to the manufacturer’s instructions. All samples and standards were analyzed in duplicate. For the evaluation of NFκB-p65 translocation, nuclear and cytoplasmic extracts were prepared using a nuclear and cytoplasmic protein extraction kit obtained from Beyotime Institute of Biotechnology (Nanjing, China).

### Statistical analysis

Data were presented as mean ± standard error of the mean. Normality of the data distributions was examined via the Kolmogorov–Smirnov test. In behavioral tests, differences among groups were analyzed by one-way ANOVA, followed by the Tukey post-hoc test. For data sets that did not conform to a normal distribution, the Kruskal–Wallis test was conducted, with Dunn’s test used for post-hoc group comparisons. The Morris water maze (MWM) training data were analyzed using repeated-measures two-way ANOVA. All statistical analyses were carried out with GraphPad Prism 7.0 software (San Diego, CA, United States). A two-tailed P - value of less than 0.05 was regarded as statistically significant.

## Results

### Effects of VTX on the brain damage of rats after MCAO/R

**FIGURE 2:**
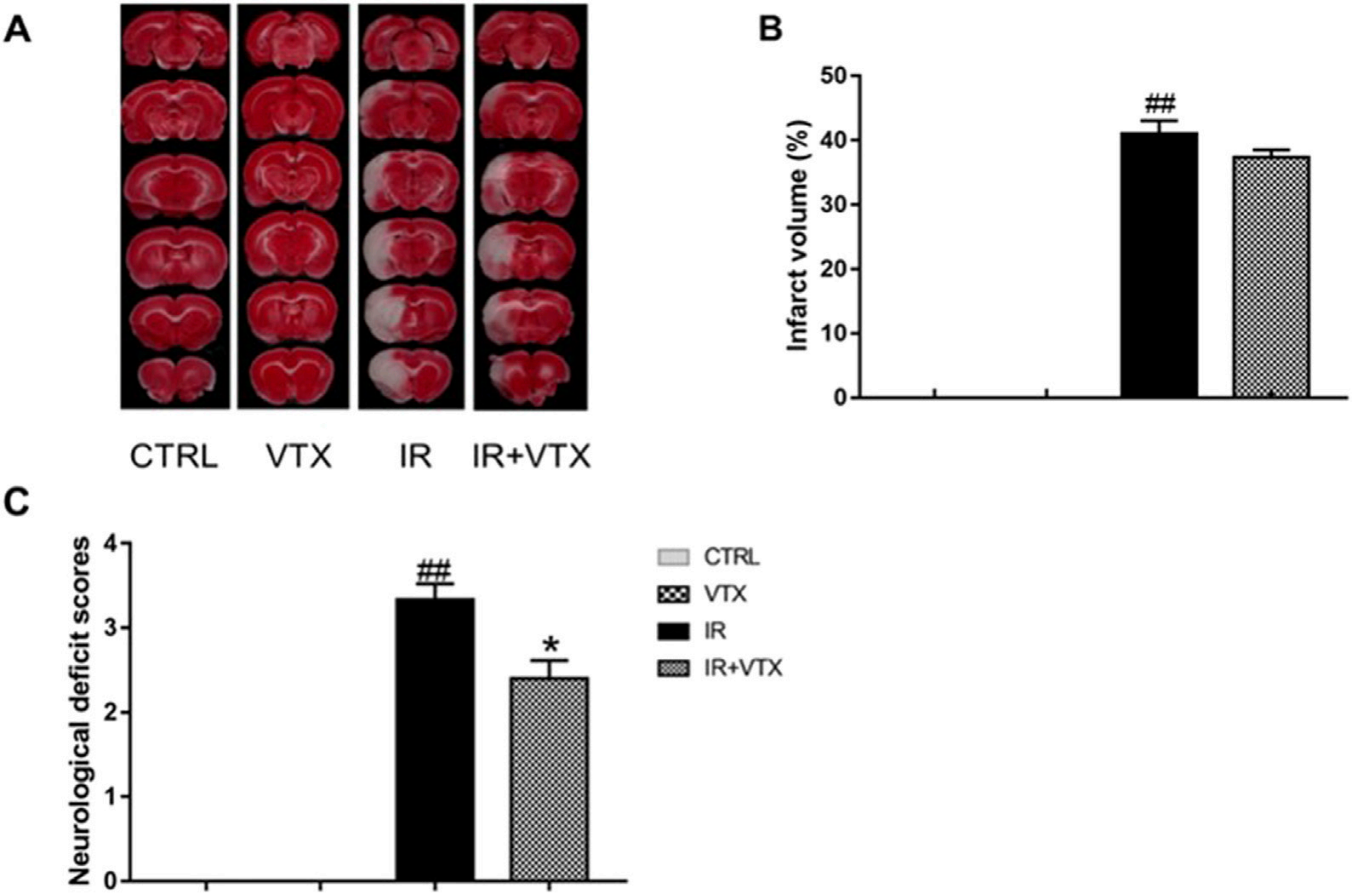
Effects of VTX on cerebral infarct size, neurological deficit scores in I/R rats. **(A, B)** Effects of VTX on the infarct area in the left hemisphere of the brains in I/R rats (n = 3). **(C)** Effects of VTX on neurological scores (n = 15). Data are expressed as the mean ± SEM and were analyzed by one-way ANOVA (*post hoc* analysis: Tukey’s *post hoc* analysis test). * (*p* < 0.05) and ** (*p* < 0.01) represent significance compared to the I/R group. # (*p* < 0.05) and ## (*p* < 0.01) represents significance compared to the CTRL group.

Two weeks after reperfusion, TTC staining was employed to assess the cerebral infarction size in rats. The percentage of infarct volume increased significantly in the IR group (*p* < 0.01) (Figures 2A, C). In addition, compared with the control group, the IR + VTX group showed a tendency for a decrease in infarct volume. Nevertheless, this difference did not achieve statistical significance (*p* > 0.05).

#### VTX enhanced neurological function of rats after MCAO/R

As depicted in Figure 2B, when compared to the CTRL group, the rats in the I/R group demonstrated evident neurological deficits (*p* < 0.01). VXT significantly improved neurological function (*p* < 0.05).

#### VTX improved the locomotor activity of rats after MCAO/R

**FIGURE 3:**
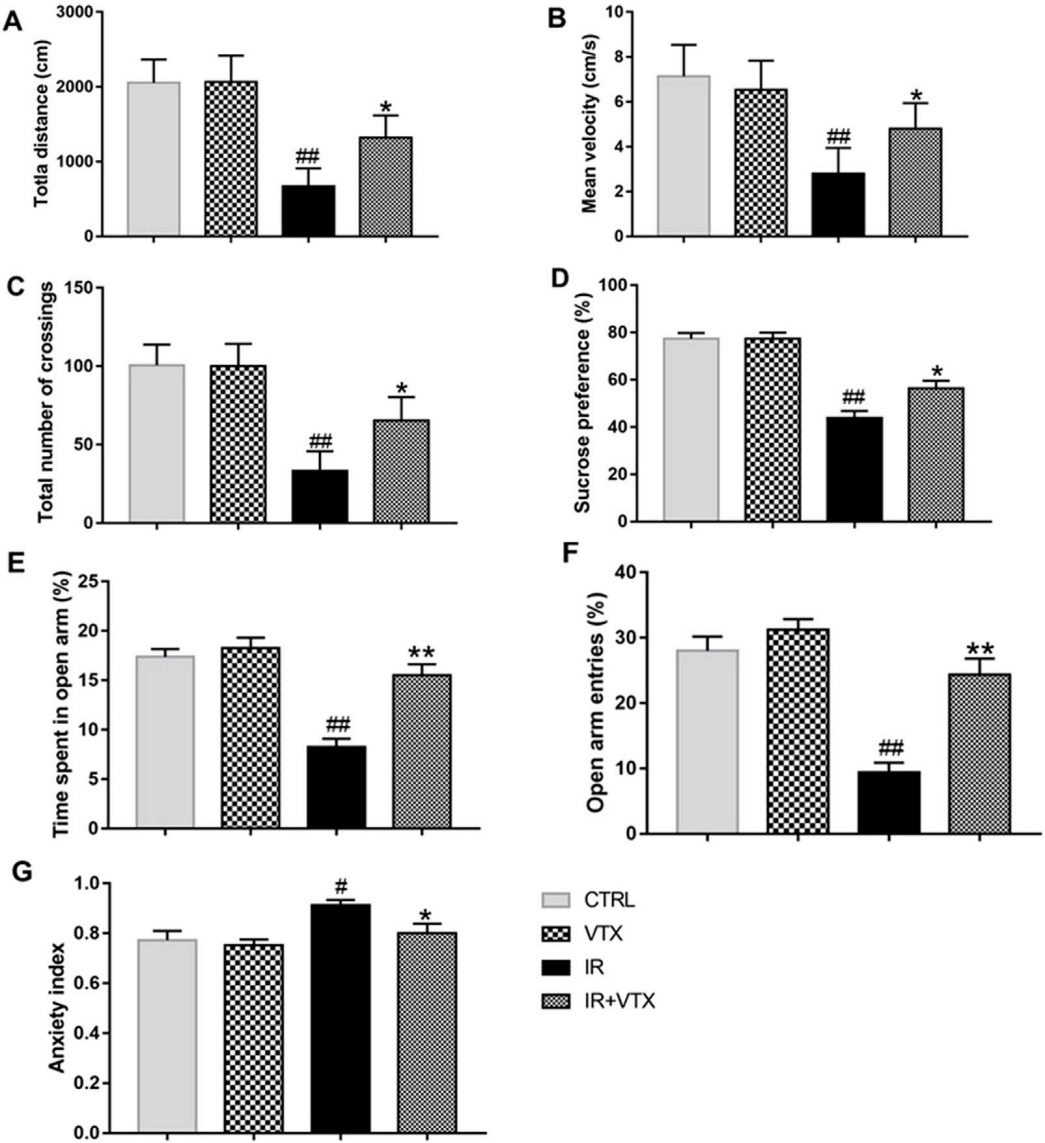
Effects of VTX on the locomotor activity, depression-like behavior and anxiety-like behavior of rats after MCAO/R. **(A–C)** Effects of VTX on locomotor activity of rats after MCAO/R. **(D)** Effects of VTX on the depression-like behavior of rats after MCAO/R. **(E–G)** Effects of VTX on the anxiety-like behavior of rats after MCAO/R. Data are expressed as the mean ± SEM (n = 12) and were analyzed by one-way ANOVA (*post hoc* analysis: Tukey’s *post hoc* analysis test). * (*p* < 0.05) and ** (*p* < 0.01) represent significance compared to the I/R group. # (*p* < 0.05) and ## (*p* < 0.01) represents significance compared to the CTRL group.

The open field test was utilized to assess motor deficits in rats following cerebral ischemia. Based on the findings, the total distance traveled, mean velocity, and the total number of square crossings in the IR group were significantly lower than those in the CTRL and VTX groups (*p* < 0.01). These scores were significantly increased by VTX treatment in IR + VTX group (*p* < 0.05), but remained lower compared to the CTRL and VTX group (Figures 3A–C).

#### VTX alleviated the depression-like behavior of rats after MCAO/R

The antidepressant effect of vortioxetine on post-stroke depression-like behavior in rats was evaluated using the sucrose preference test (Figure 3D). During the adaptation period (the first 24 h of the SPT), no significant difference existed between the groups. However, in the 48-hour sucrose preference test, there was a significant difference in sucrose preference between the groups. Compared with the CTRL and VTX groups, the sucrose preference of the IR rats was significantly reduced (*p* < 0.01). Treatment with vortioxetine observably increased sucrose preference (*p* < 0.05).

#### VTX ameliorated anxiety-like behavior in rats after MCAO/R

Anxiety-like behaviors were assessed by EPM tests. In comparison with the CTRL group, the IR group showed a substantial decrease in both the time spent and the frequency of entries into the open arms (*p* < 0.01). The IR + VTX group demonstrated a marked increase in the duration and frequency of open arm entries (*p* < 0.01), along with a significant decrease in the anxiety index (*p* < 0.05), suggesting that VTX can alleviate stroke-induced anxiety-like symptoms in rats (Figures 3E–G).

#### VTX improved the learning and memory abilities of rats after MCAO/R

**FIGURE 4:**
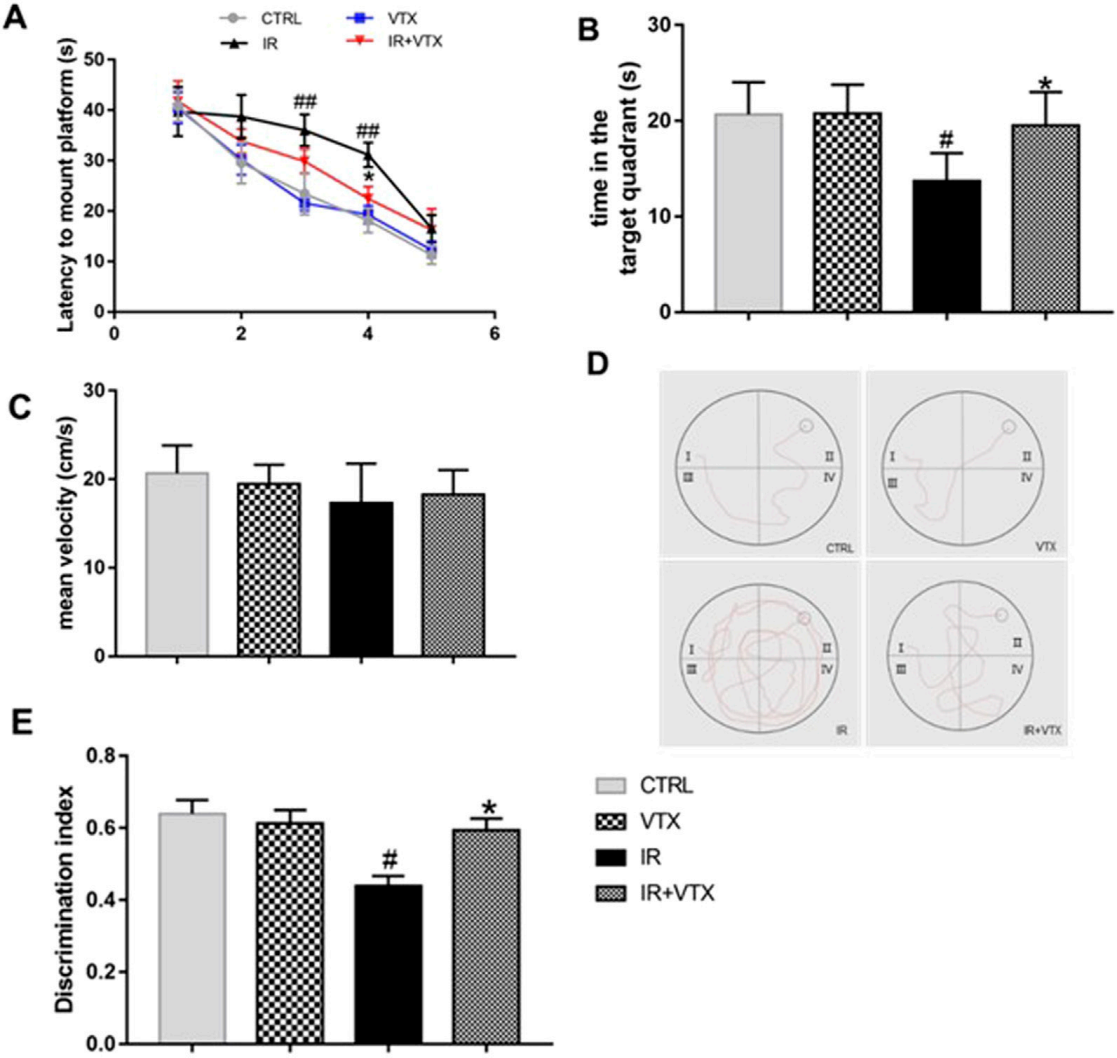
Effects of VTX on the cognitive impairments of rats after MCAO/R. **(A–D)** VTX ameliorates spatial reference learning and memory deficits after MCAO/R. **(A)** The time of latency to mount platform. During the hidden platform training, as the number of training days increased, the time required to find the platform decreased. **(B)** The time spent in the target area on probe day. **(C)** The average speed of the four groups during the free swimming phase. **(D)** Representative recorded track plot during training period. **(E)** Discrimination index during the novel object recognition task. Data are expressed as the mean ± SEM (n = 12), the time spent in the target area on probe day were analyzed by two-way ANOVA, the rest of data were analyzed by one-way ANOVA. * (*p* < 0.05) and ** (*p* < 0.01) represent significance compared to the I/R group. # (*p* < 0.05) and ## (*p* < 0.01) represents significance compared to the CTRL group.

To confirm the impaired cognitive functions after MCAO/R, the MWM test was performed. In the 5-day training period, the time spent by ischemia-reperfusion (IR) rats before reaching the platform was longer than that of the CTRL group (Figure 4A). The learning pattern and time of all groups of rats were negatively correlated during the training period. However, there were significant differences in the average escape latency and total distance moved between different experimental groups. Compared with the CTRL group, the IR group had a longer escape latency on day 3 and 4 of training (*p* < 0.01). Treatment with VTX significantly reduced the escape latency of IR rats on the fourth days (*p* < 0.05). During the fifth day of training, the escape latency showed a tendency to decline, and no significant difference was observed among the groups (Figure 4A). Moreover, throughout the training period, no significant difference was detected between the CTRL and VTX groups (*p* > 0.05).

On the sixth day, a spatial exploration test was conducted to evaluate the spatial memory of the rats. The results showed that the time spent by the IR rats in the target quadrant was less than that of the CTRL group (*p* < 0.05). Treatment with VTX significantly increased the time spent by IR rats in the target area (*p* < 0.05) (Figure 4B). No significant difference was found between the CTRL and VTX groups. At the same time, we calculated the swimming speed of all rats to evaluate whether the damaged motor function would affect the evaluation of learning and memory ability in MWM. The results indicated that there was no significant difference in swimming speed among the groups (Figure 4C). The path of rats searching for the hidden platform during the learning period is shown in the Figure 4D. Video tracking software was employed to acquire these images on the fourth day of the experiment for each group. A black circle denotes the platform. Evidently, in the experiment, rats in the IR group mostly swam in incorrect quadrants and took a longer time to locate the platform. In contrast, rats in the IR + VTX group could remarkably shorten the time it took to find the platform (*p* < 0.05).

Furthermore, the novel object recognition test is a validated method to assess the memory ability of rats after MCAO/R. The results of the experiment showed that the IR group had a significantly lower recognition index than the CTRL and VTX groups (*p* < 0.05). However, treatment with VTX significantly improved the exploratory ability of rats towards new objects (*p* < 0.05), suggesting that VTX has the potential to enhance the learning and memory capabilities of rats following cerebral ischemia (Figure 4E).

### Effects of VTX on modulation of pro-inflammatory cytokines and NF-*κ*B p65 in the hippocampus and prefrontal cortex after MCAO/R

**FIGURE 5:**
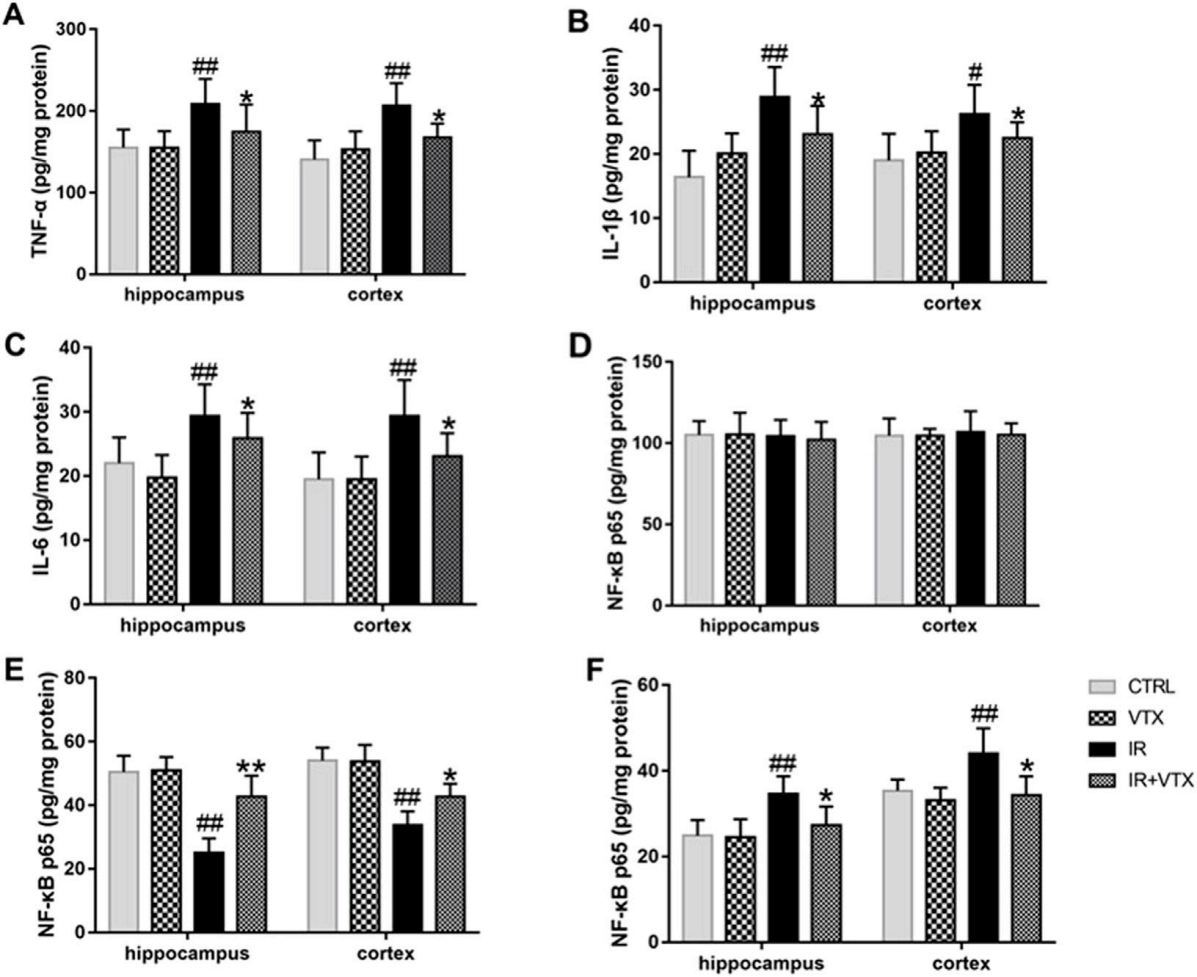
Effects of VTX on Modulation of pro-inflammatory cytokines and NF-*κ*B p65 in the hippocampus and prefrontal cortex after MCAO/R. **(A–C)** The levels of pro-inflammatory cytokines (TNF-ɑ, IL-1β, IL-6) in the hippocampus and prefrontal cortex after MCAO/R. **(D–F)** Effect of VTX on NF-*κ*B p65 activation after MCAO/R. NF-*κ*B p65 protein secretion levels in whole cells **(D)**, cytosolic **(E)**, and nuclear **(F)** were analyzed by ELISA. Data are expressed as the mean ± SEM (n = 3) and were analyzed by one-way ANOVA (*post hoc* analysis: Tukey’s *post hoc* analysis test). * (*p* < 0.05) and ** (*p* < 0.01) represent significance compared to the I/R group. # (*p* < 0.05) and ## (*p* < 0.01) represents significance compared to the CTRL group.

To elucidate the regulatory effects of VTX on post-stroke neuroinflammation in rats, we measured the levels of pro-inflammatory cytokines and NF-*κ*B p65 in the hippocampus and prefrontal cortex using ELISA. The present research demonstrated that in the IR group, the levels of pro - inflammatory cytokines (TNF - ɑ, IL - 1β, and IL - 6) in the hippocampus and cortex were markedly elevated (*p* < 0.01). In addition, the levels of pro-inflammatory cytokines in these two regions were significantly decreased in the IR + VTX group compared with the IR group (*p* < 0.05) (Figures 5A–C).

To better clarify the possible molecular mechanism via which VTX curbs neuroinflammatory responses, we observed the activation of the transcription factor NF-*κ*B. The activation of NF-*κ*B happens as a response to pro - inflammatory stimuli and brings about an increase in the expression of a variety of cytokines and chemokines. There was no significant difference in whole-cell NF-*κ*B p65 protein levels in hippocampus and cortex (*p* > 0.05). However, cytoplasmic NF-*κ*B-p65 protein levels were significantly reduced in the hippocampus and cortex in the IR group (*p* < 0.05), while nuclear protein levels were significantly increased in both brain regions (*p* < 0.01) (Figures 5D–F), indicating that NF-*κ*B p65 was activated and transferred to the nucleus. Administration of VTX demonstrates reversal of NF-κB p65 translocation, hinting at its capacity to suppress neuroinflammation by curtailing NF-κB activation. This action elucidates a pivotal mechanism underlying VTX’s neuroprotective attributes, showcasing its potential to mitigate inflammatory cascades within neuronal environments.

#### VTX attenuate the NF-*κ*B p65 and TLR-2 expression in hippocampus and prefrontal cortex of rats after MCAO/R

**FIGURE 6:**
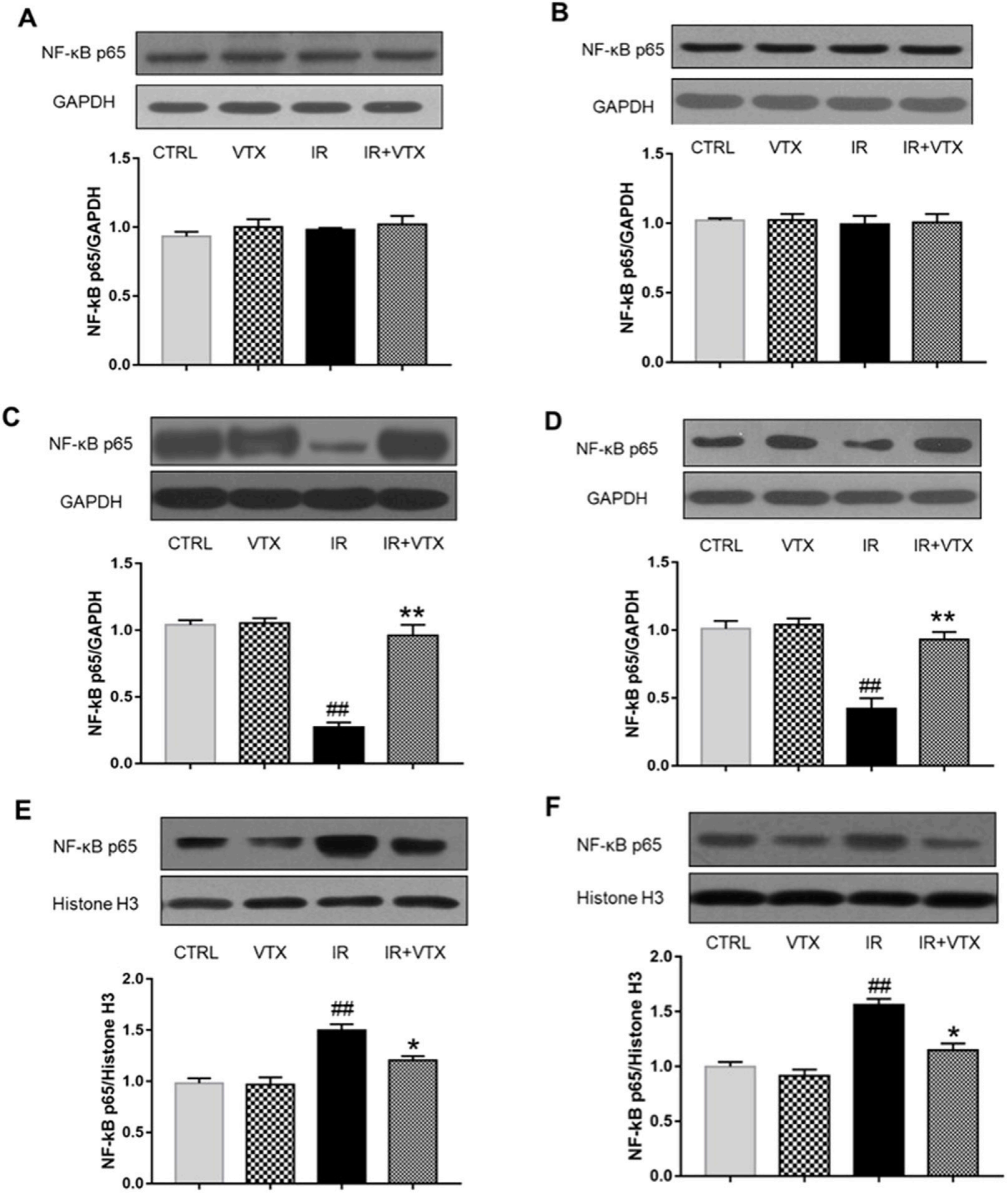
Effects of VTX on the NF-*κ*B p65 protein expression in hippocampus and prefrontal cortex of rats after MCAO/R. **(A, B)** NF-*κ*B p65 protein secretion levels in whole cells, **(C, D)** cytosolic, and **(E, F)** nuclear were analyzed by Western blot. Data are expressed as the mean ± SEM (n = 3) and were analyzed by one-way ANOVA (*post hoc* analysis: Tukey’s *post hoc* analysis test). * (*p* < 0.05) and ** (*p* < 0.01) represent significance compared to the I/R group. # (*p* < 0.05) and ## (*p* < 0.01) represents significance compared to the CTRL group.

In order to further clarify the anti-neuroinflammatory effect of VTX, we applied Western blot analysis to determine the protein expression levels of NF-*κ*B p65 and TLR-2 in the hippocampus and prefrontal cortex of rats. In accordance with the previous findings, the cytoplasmic expression level of NF-*κ*B p65 protein in IR rats was markedly reduced (*p* < 0.01), whereas its nuclear expression was markedly elevated (*p* < 0.05). These changes were significantly attenuated following VTX administration (Figures 6A–F).

**FIGURE 7:**
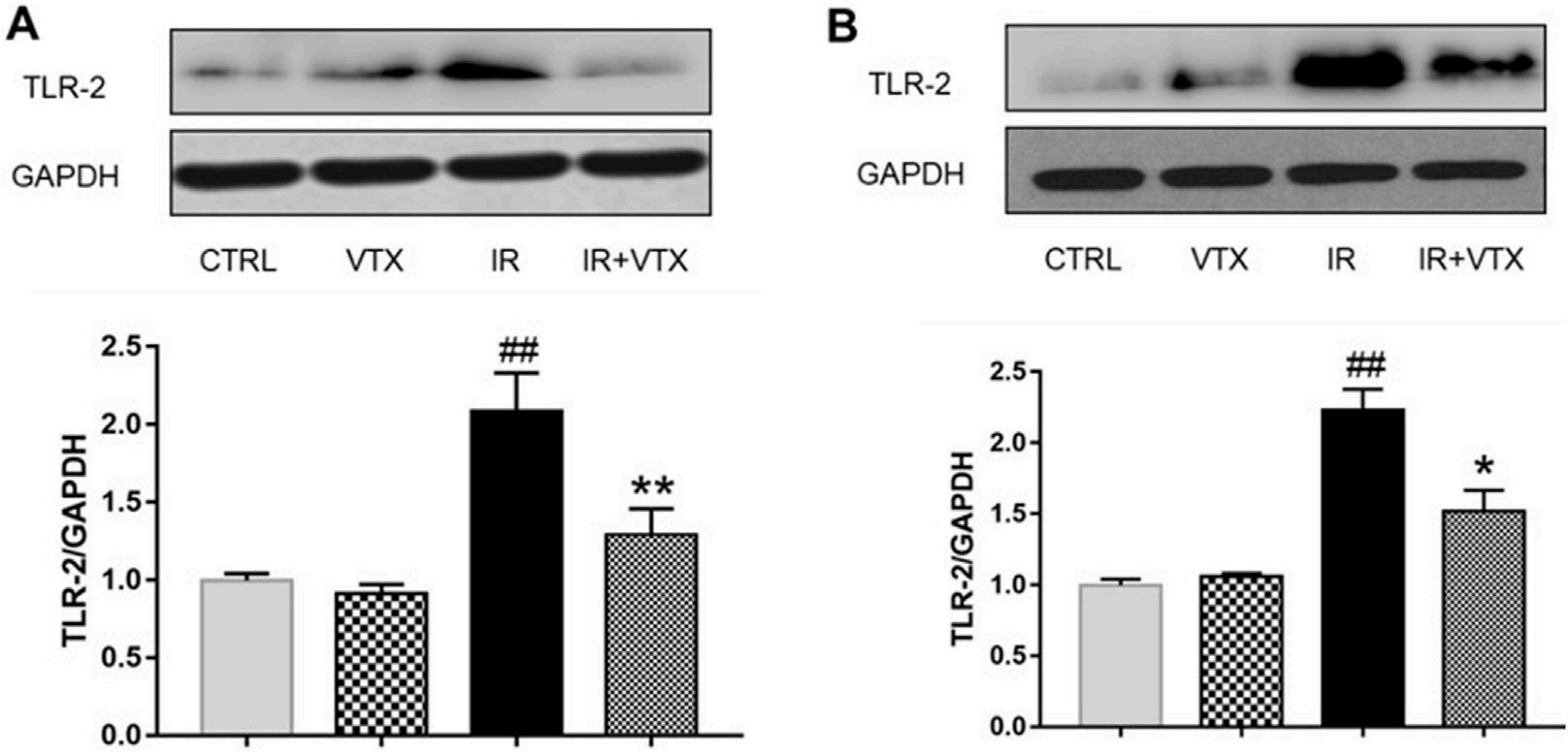
Effects of VTX on the TLR-2 protein expression in hippocampus and prefrontal cortex of rats after MCAO/R. **(A)** TLR-2 protein expression levels in hippocampus, **(B)** TLR-2 protein expression levels in prefrontal cortex were analyzed by Western blot. Data are expressed as the mean ± SEM (n = 3) and were analyzed by one-way ANOVA (*post hoc* analysis: Tukey’s *post hoc* analysis test). * (*p* < 0.05) and ** (*p* < 0.01) represent significance compared to the I/R group. # (*p* < 0.05) and ## (*p* < 0.01) represents significance compared to the CTRL group.

The findings indicated that, when compared with the CTRL group, the levels of TLR - 2 in the hippocampus and prefrontal cortex of rats in the IR group were remarkably increased (*p* < 0.01), while the TLR-2 levels in the hippocampus and prefrontal cortex of rats in the IR + VTX group were significantly decreased compared to the IR group (*p* < 0.05) (Figures 7A, B).

## Discussion

Stroke represents a grave menace to global health, manifesting debilitating sequelae such as hemiparesis, speech impairment, localized neurological deficits, and concurrent depressive behaviors, often coupled with compromised learning-memory and cognitive abilities. Despite life-saving therapies, remediation of cognitive dysfunction remains elusive (Robinson and Jorge, 2016; Frank et al., 2022). Emerging data suggests antidepressants’ potential in alleviating post-stroke depression-like behaviors and augmenting cognitive performance across various stroke models (Roman et al., 2010; Auchus et al., 2007; Medeiros et al., 2020). Notably, their neuroprotective actions might be mediated partly through anti-inflammatory pathways. VTX, a multi-modal serotonergic antidepressant, touts cognitive-enhancing traits exerted directly or indirectly on diverse neurotransmitter systems. Hence, our study endeavors to probe VTX’s influence on motor coordination, cognitive function, and depressive behaviors within a Middle Cerebral Artery Occlusion (MCAO) rat paradigm, while dissecting possible mechanistic underpinnings.

Following acute ischemic stroke, disruption of cerebral blood flow engenders ischemia and hypoxia, precipitating tissue necrosis and neuronal damage, culminating in motor and cognitive impairments. Our outcomes underscore VTX’s capacity to alleviate neurological deficits triggered by global ischemic-reperfusion trauma, evidenced by diminished neurological severity scores within the Ischemia-Reperfusion plus VTX (IR + VTX) cohort relative to the IR-only group. Such observations align with precedent investigations illustrating VTX’s neuroprotective benefits across distinct cerebral ischemia models (Martis et al., 2021; Nemutlu et al., 2022). Notwithstanding, we discern limitations: VTX’s inability to fully reverse injuries indicates a constrained therapeutic scope vis-a-vis acute brain trauma. Intriguingly, our analysis revealed a trend toward reduced infarct size in the IR + VTX cohort versus controls, albeit lacking statistical significance. These findings are consistent with a previous study that demonstrated the potential protective effect of VTX on cerebral infarction after ischemia reperfusion injury (Emam et al., 2021). Collectively, our study reinforces VTX’s neuroshielding effects against acute cerebral injury, substantiating its prospective clinical utility in managing acute brain pathologies.

Stroke, a prevalent neurological ailment, drastically curtails autonomy, impacting mobility via multifaceted mechanisms encompassing motor cortex impairment, disrupted neural connectivity, muscular atrophy, and attenuated neuroplasticity (Zhao et al., 2022). Precedent literature posits antidepressants’ capability to enhance post-stroke rodent locomotion, positively influencing cerebral infarction patient outcomes (Paolucci, 2017). Within our open-field testing framework, VTX notably augmented spontaneous activity in stroke-afflicted rats, heralding potential motor recuperative benefits post-stroke.

Cognitive dysfunction ranks as the paramount post-stroke complication, afflicting over half of stroke survivors, manifesting as declines in attention, executive function, memory, visuospatial acumen, and linguistic proficiency. Underlying mechanisms implicate lesions in cognitive domains and circuitries, including the hippocampus, prefrontal cortex, and striatal networks (Tater and Pandey, 2021). Research posits that posterior cerebral artery ischemia-induced hippocampal harm instigates cognitive deterioration and enduring memory deficits (Osborne et al., 2024).

Our investigation probed VTX’s impact on post-stroke cognition in rats, employing novel object recognition and Morris Water Maze (MWM) assessments. Novel object recognition outcomes delineated markedly impaired recall among ischemia-reperfusion (IR) subjects juxtaposed with Control and VTX cohorts. Strikingly, VTX administration bolstered rats’ investigative prowess towards unfamiliar items, indicative of augmented learning and memory faculties post-cerebrovascular accident.

The MWM test revealed that IR rats had longer escape latency and total distance traveled during the training period compared to CTRL and VTX groups. However, early treatment with VTX significantly reduced the escape latency and total distance traveled by IR rats, indicating that VTX can improve spatial learning and memory in rats after stroke. Moreover, the spatial exploration test on the sixth day showed that IR rats spent less time in the target quadrant than CTRL rats, while treatment with VTX significantly increased the time spent by IR rats in the target quadrant.

Overall, these findings provide compelling evidence that VTX can enhance cognitive function in rats after stroke, without confounding motor impairment. The results suggest that VTX may have therapeutic potential for the treatment of cognitive impairment associated with stroke in humans, although further studies are needed to confirm these findings.

Post-stroke depression is a common complication with an incidence of 30%–50% (Medeiros et al., 2020). Its occurrence is related to age, gender, stroke type, severity of disease, social support and other factors, and is closely related to inflammation (Anrather and Iadecola, 2016). In terms of prevention and treatment, exercise and psychological intervention can play a role (Pandian et al., 2018). Future studies should explore its pathogenesis more deeply and develop more effective prevention and treatment methods to improve patients’ quality of life. VTX improved the sucrose preference of post-stroke rats, indicating an anti-poststroke depression effect. This finding is consistent with previous studies that have reported the antidepressant effects of VTX in animal models of depression (Christensen et al., 2023; Sanchez et al., 2015).

Consistent with prior research, the IR group exhibited a significant decrease in the duration and frequency of entries into the open arms, whereas the IR + VTX group showcased a marked increase in both parameters alongside a notable reduction in anxiety index (p < 0.05), suggesting that VTX can alleviate anxiety-like symptoms induced by stroke (Adair et al., 2023; Singh et al., 2024).

Cerebral ischemia triggers diffusion impairment of the blood-brain barrier, concomitant with an accumulation of pro-inflammatory mediators (TNF-α, IL-1β, IL-6) within the brain and serum, provoking an inflammatory cascade. Pro-inflammatory cytokine modulation in discrete neural territories, notably the hippocampus and prefrontal cortex, has garnered considerable attention as diagnostic indices and predictors of therapeutic efficacy in post-stroke depressive disorder. Of note, hippocampal and prefrontal cortical inflammatory perturbations steer behaviors linked to cognitive performance, underpinning the interplay between inflammation, cognitive malfunction, and depression onset (Harrison et al., 2015; Perry et al., 2007). Corroborating these insights, our research unveiled heightened TNF-α, IL-1β, and IL-6 concentrations in hippocampal and prefrontal cortical tissues post-cerebral ischemia. Conversely, VTX pretreatment markedly suppressed inflammatory mediator expression in these key regions. Consequently, VTX administration alleviated motor, cognitive, and affective dysfunctions in acute cerebral infarction-stricken rats, seemingly mediated via neuroinflammation attenuation.

Recent studies have reported increased expression of TLR-2 in the hippocampus of rats with acute cerebral ischemia (Monaco et al., 2009). Consistent with our experimental results, TLR2 receptor levels in the hippocampus and prefrontal cortex of MCAO rats were significantly increased. As expected, because of the drug’s potential anti-inflammatory properties, VTX treatment reduced TLR-2 accumulation in the hippocampus and prefrontal cortex, suggesting that VTX’s inhibitory effect on neuroinflammation is most likely mediated by inhibition of TLR-2 receptors. The molecular mechanism by which vortioxetine inhibits TLR-2 may involve its multimodal receptor interaction (Stahl, 2015). It may be through the inhibition of microglia activation, which is a key source of TLR-2 overexpression in cerebral ischemia. It has also been pointed out that vortioxetine enhances CREB/BDNF signaling, thereby counteracting NF-κB-driven TLR-2 transcription (Shafiek et al., 2024).

Our investigation further quantified NF-*κ*B p65, a pivotal node in the TLR-2 signaling axis, intimately tied to inflammatory processes (Nemutlu et al., 2022). As a transcription factor, the elevated level of NF-*κ*B is associated with neuroinflammation and neurodegeneration, and is an important regulator of the transcription of these inflammatory cytokines. Activation of NF-*κ*B stimulates the expression of pro-inflammatory cytokines, thus exacerbating inflammation, while inhibition of the activation of NF-*κ*B inhibits the expression of various genes, such as TNF-ɑ, IL-1β and IL-6. Upon microglial activation, NF-*κ*B translocates from cytoplasm to nucleus, orchestrating immune responses (Zou et al., 2017). Strikingly, VTX pretreatment effectively blunted NF-*κ*B activation, insinuating that VTX’s anti-inflammatory properties might hinge upon NF-*κ*B suppression, ostensibly modulated by TLR2 receptors.

## Conclusion

In summation, this study scrutinized VTX‘s impacts on motor competence, anxiety, spatial cognition, and depression-like conduct in MCAO rodent models. Despite not wholly reversing ischemia-reperfusion pathology, VTX demonstrably enhances rats’ motor agility, augments post-stroke learning-memory aptitude, and exerts antidepressant-anxiolytic actions. Current data propose VTX’s salubrious influences on mobility, cognition, and mood disorders, potentially attributable to its anti-inflammatory virtues, perhaps via TLR-2/NF-*κ*B axis suppression. While these preclinical findings highlight vortioxetine’s therapeutic potential, several limitations warrant consideration. First, rodent models cannot fully replicate human stroke pathophysiology, particularly regarding comorbid conditions and long-term recovery trajectories. Second, the selected dose (10 mg/kg) corresponds to human-equivalent antidepressant doses but may not reflect optimal dosing for neuroprotection. Future studies should explore dose-response relationships across recovery phases and validate TLR-2/NF-*κ*B pathway modulation in human biomarker studies. Translational efforts could prioritize post-stroke depression patients with elevated inflammatory markers, where vortioxetine’s dual mechanisms may offer maximal benefit.

## Data Availability

The original contributions presented in the study are included in the article/supplementary material, further inquiries can be directed to the corresponding author.
